# Supplementary material for: Ircinia ramosa Sponge Extract (iSP) Induces Apoptosis in Human Melanoma Cells and Inhibits Melanoma Cell Migration and Invasiveness
Source: Mar Drugs. 2023 Jun 24;21(7):371. doi: 10.3390/md21070371 (PMC10381260; doi:10.3390/md21070371)
Supplement: Supplementary file 1 [file marinedrugs-21-00371-s001.zip › marinedrugs-2430334-supplementary.pdf]

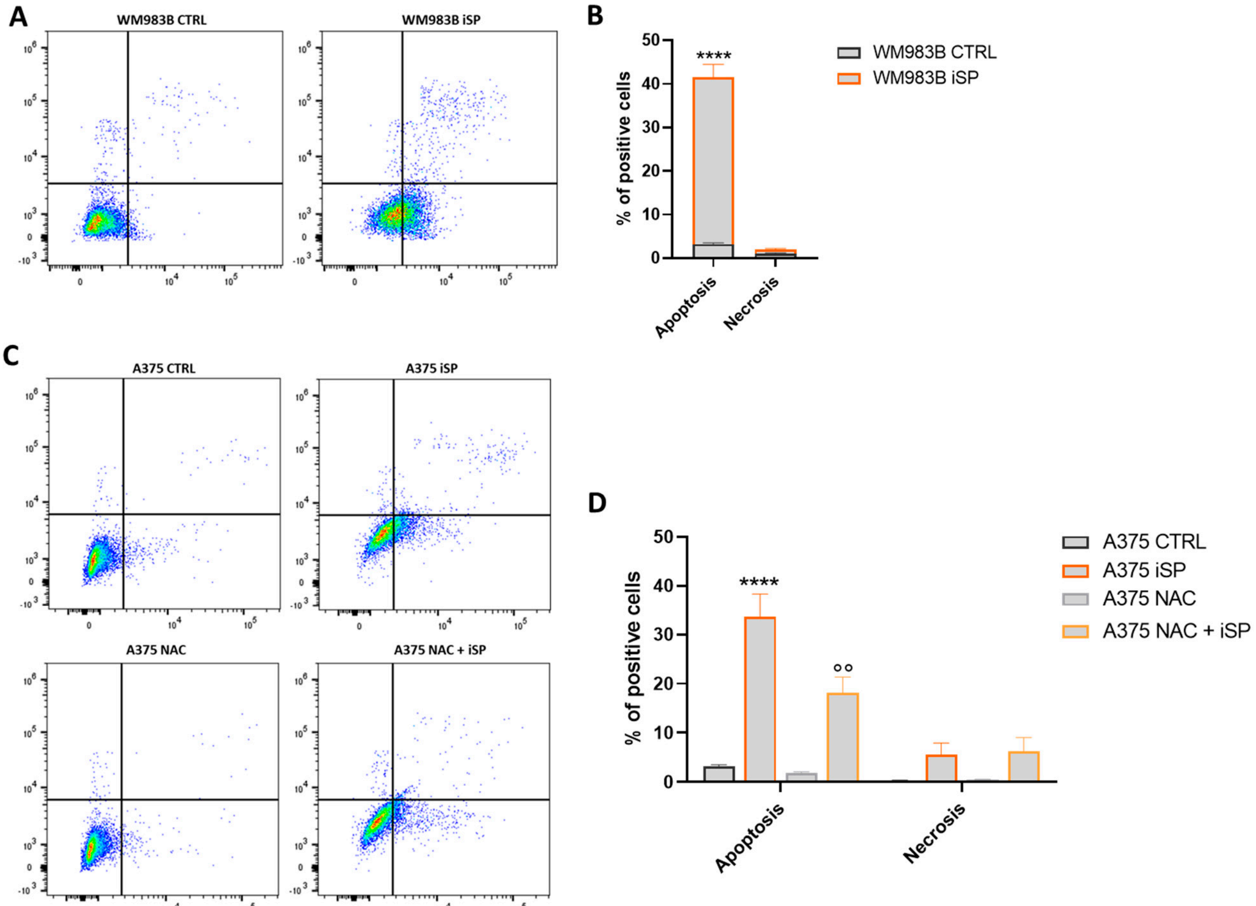

Supplementary Figure S1. A) Representative example of annexin V/propidium iodide (PI) staining after 48 hr treatment or not of WM983B cells with iSP 30 μg/ml. B) Frequency of apoptotic WM983B cells after treatment (orange bar) or not (black bar) for 48 hr with iSP 30 μg/ml. C) Representative example of annexin V/propidium iodide (PI) staining after 48 hr treatment or not of WM983B cells with iSP 30 μg/ml and preincubated or not with NAC 2 mM for 1 hr. D) Frequency of apoptotic cells after incubation (grey bar) or not (black bar) with NAC and treatment for 48 hr with iSP 30 μg/ml (orange and light orange bars). Data are shown as mean ± SEM of at least three independent experiments (**** $p < 0.01$ vs. A375 CTRL; °° $p < 0.01$ vs. A375 iSP).
